# Steam Explosion-Assisted Extraction of Protein from Fish Backbones and Effect of Enzymatic Hydrolysis on the Extracts

**DOI:** 10.3390/foods10081942

**Published:** 2021-08-20

**Authors:** Ye Dong, Wen Yan, Xiao-Di Zhang, Zhi-Yuan Dai, Yi-Qi Zhang

**Affiliations:** Collaborative Innovation Center of Seafood Deep Processing, Key Laboratory of Aquatic Products Processing of Zhejiang Province, Institute of Seafood, Zhejiang Gongshang University, Hangzhou 310035, China; dy12686@163.com (Y.D.); ywseafood@126.com (W.Y.); zhangxiaodixdz@163.com (X.-D.Z.); dzy@zjgsu.edu.cn (Z.-Y.D.)

**Keywords:** fish backbone, protein, steam explosion, hot-pressure extraction, enzymatic hydrolysis

## Abstract

The development of an efficient pretreatment, prior to enzymatic hydrolysis, is a good strategy for the sustainable use of refractory fish byproducts. This study compared hydrothermal pretreatments at 159 °C for 2 min, followed by water extraction (steam explosion-assisted extraction, SE) and 121 °C for 70 min (hot-pressure extraction, HPE), for the recovery of proteins from fish backbones. The effect of enzymatic hydrolysis on the properties of the obtained fish bone protein (FBP) was also evaluated. The results demonstrated that FBP had high contents of protein (81.09–84.88 g/100 g) and hydroxyproline (70–82 residues/1000 residues). After hydrolysis with Flavourzyme, for 3 h, the FBP hydrolysates that were pretreated with SE (SFBP-H) exhibited a better degree of hydrolysis (DH) and nitrogen recovery (NR), and a higher level of umami taste free amino acids (151.50 mg/100 mL), compared with the HPE-treated samples. The obtained SFBP-H mainly distributed below 3000 Da and had strong scavenging effects on 1,1-diphenyl-2-picrylhydrazy (DPPH) (IC_50_ = 4.24 mg/mL) and 2,2-azino-bis(3-ethylbenzothiazoline-6-sulphonic acid) (ABTS) (IC_50_ = 1.93 mg/mL) radicals. Steam explosion-assisted extraction is a promising route for recovering proteins from native fish bone materials, and improving the flavor and antioxidant activity of the hydrolysates.

## 1. Introduction

Bighead carp (*Aristichthys* *nobilis*) is one of the common freshwater fish species in China; approximately 3.1 million tons were cultured in 2019 [1]. During fish fillet and bighead carp head processing, large amounts of fish byproducts are inevitably generated, including about 9% to 11% bone and fin, 7% to 9% viscera, and 1% to 5% scales, with respect to the total body weight [2]. These fish byproducts, with low economic value, are usually treated as waste, leading to environmental problems. Particularly, fish bones are good sources of protein and bio-calcium [3]. After appropriate processing, these raw materials may find potential applications among a series of food products [4,5,6,7].

Traditionally, collagen can typically be solubilized and extracted from native collagen materials, by highly concentrated acid or alkali treatments, over a long period [8]. Although acidic or alkaline hydrolysis has been used for protein recovery from fish byproducts [9,10], these methods are generally reactant, cost, and time consuming, and even bad for the environment [11]. Recently, enzymatic hydrolysis, a mild and controllable method, has been widely used for the extraction of proteins and the production of protein hydrolysates from fish-processing industries [12,13]. However, the efficiency of the hydrolysis process for fish bone is very low, due to the complex bony structure’s resistance to commercial proteases [3]. Overall, it is still difficult to completely extract proteins from the bone materials by enzymatic hydrolysis [14]. Therefore, the development of a green and effective method for the recovery of proteins from native bone materials, and the efficient release of low-molecular-weight peptides, seems justifiable.

The modification of protein structure, through innovative pretreatment prior to enzymatic hydrolysis, is a good strategy to enhance the hydrolysate properties [15]. Recently, the ability of hydrothermal processing (about 120–250 °C), such as hot-pressure extraction [14] and subcritical water hydrolysis [16], in the hydrolysis of bio-macromolecules, has been explored. Under hydrothermal conditions, the ionic product of water is increased, through the formation of hydronium and hydroxide ions, making water or steam appear as an acid or base catalyst [17]. In recent years, hydrothermal pretreatment, with or without enzymatic hydrolysis, has been used to extract protein and produce protein hydrolysates or free amino acids from different protein sources, including chicken bone [14], porcine skin [18], and fish byproducts [16,19,20,21,22].

Steam explosion is an innovative physicochemical process, involving the quick treatment of the biomass under saturated steam pressure (0.5–2.0 MPa, about 150–210 °C), followed by explosive decompression (within 0.00875 s) and instant temperature reduction to 50 °C or lower [23]. Due to the lower environmental impact and energy consumption, it has been applied in the pretreatment of raw materials, such as native lignocellulosic materials and agricultural byproducts [24]. Except for the thermal effect, as in traditional hydrothermal treatment, there is also a physical tearing effect that is accompanied by the rapid pressure release during the steam explosion process [24]. This process can overcome the key barriers limiting degradation, thereby increasing extraction and the enzyme accessibility of the biomass. Steam explosion equipment, with a 10 m^3^ biomass chamber, has been developed [23], increasing its potential for industrial applications. In a recent study, Shen et al. used steam explosion treatment to liquefy chicken sternal cartilage for chondroitin sulfate isolation [25]. Previously, we developed a steam explosion-assisted dissolution process, to extract keratin from feathers [26]. Guo et al. used steam explosion-assisted conventional protease to hydrolyze feathers, eliminating the need for substrate-specific keratinases [23]. Thus, we hypothesized that the steam explosion could be an efficient alternative for extracting protein from fish backbones and improving the accessibility of bone protein extracts to commercial proteases. The present work is the first use of steam explosion-assisted extraction, for the recovery of proteins from fish backbones. 

The aim of this study was to develop a method for the recovery of proteins from fish backbones, and to investigate the effect of enzymatic hydrolysis on the recovered extracts. In this work, the effects of steam explosion-assisted extraction and hot-pressure extraction on protein recovery, degree of hydrolysis, amino acid composition, structural properties, and antioxidant activity of fish bone protein and its hydrolysates, were compared. The findings may help to promote more effective utilization of refractory byproducts that are generated during fish processing.

## 2. Materials and Methods

### 2.1. Materials and Reagents

Fresh bighead carp backbones were cut into segments of about 6 cm and stored at −20 °C. Frozen backbones were thawed at 10 °C before use. Flavourzyme was purchased from Novozyme (Tianjin, China). Cytochrome C, aprotinin, bacitracin and hippuryl-histidyl-leucine (HHL) were obtained from Sigma-Aldrich (Milwaukee, WI, USA). Further, 2,2-Azino-bis(3-ethylbenzothiazoline-6-sulphonic acid) (ABTS) diammonium salt and 1,1-diphenyl-2-picrylhydrazy (DPPH) were obtained from Aladdin Reagents Co., Ltd. (Shanghai, China). Acetonitrile (ACN) and trifluoroacetic acid (TFA) were of chromatographical grade. All the other chemicals were of analytical grade and were purchased from Sinopharm Chemical Reagent Co., Ltd. (Shanghai, China).

### 2.2. Preparation of Fish Bone Protein

Fish bone proteins (FBP) were prepared through steam explosion-assisted extraction (SE) and hot-pressure extraction (HPE) processes, respectively. The steam explosion pretreatment was performed on a steam explosion device (QBS-200B, Gentle Science & Technology Co. Ltd., Hebi, China). About 300 g of fish backbones were put into a 5 L material vessel and exposed to the saturated steam at 159 ± 0.5 °C for 2 min, and finally, were terminated by explosive decompression. The exploded materials were collected and ground with an IKA A11 basic analytical mill (IKA, Staufen, Germany) to pass through a 0.38-mm-diameter mesh, and then extracted in distilled water (1:3, *w*/*v*) at 60 °C for 2 h in a water bath. In another set of experiments, fish backbones were further cut into segments of about 0.5 cm, mixed with three volumes of distilled water (*w*/*v*) and hanged in an extraction pot at 121 ± 0.5 °C for 70 min through HPE process according to Tan et al. [20]. The resulting mixtures were both filtered using gauze to remove the backbone residues. The filtrates were collected, freeze-dried with lyophilizer (Freezone 2.5 L, Labconco, Kansas City, MO, USA) and then stored at −20 °C until further analysis. The fish bone protein powder prepared by SE and HPE pretreatment was designated as SFBP and HFBP, respectively.

### 2.3. Preparation of Bone Protein Hydrolysates

The FBP dispersions (3%, *w*/*v*) were hydrolyzed using Flavourzyme with an enzyme/substrate ratio of 2% (*w*/*w*) under optimum conditions (50 °C and pH 7.0) at 125 rpm in a rotary thermostatic oscillator. The hydrolysates were taken at 0, 0.5, 1, 2 and 3 h, respectively, heated immediately at 95 °C for 15 min to inactive the enzyme and then centrifuged (Hettich Rotina 420R, Tuttlingen, Germany) at 8000× *g* for 15 min. The supernatant was collected and stored at −20 °C for further analysis.

### 2.4. Chemical Analysis of FBP

The protein, moisture, fat and ash contents of SFBP and HFBP were determined by the AOAC methods [27]. 

### 2.5. Determination of the Degree of Hydrolysis

The degree of hydrolysis (DH) of the hydrolysates was analyzed according to Tan et al. [20] with some modifications. In this study, 5 mL of hydrolysate supernatant, at different times, was mixed with 60 mL of distilled water, and the pH value of the mixture was adjusted to 8.2 using 0.05 M NaOH, followed by addition of 10 mL of neutral formaldehyde solution. The titration with the alkali solution was continued to the end point of pH 9.2 and the accurate consumed volume of 0.05 M NaOH was measured to determine the DH. 

### 2.6. Determination of Nitrogen Recovery

The nitrogen content of the supernatant and total nitrogen in the samples was measured by the Kjeldahl method. Nitrogen recovery (NR) was expressed as the soluble nitrogen in the supernatant divided by the total nitrogen in FBP.

### 2.7. Fourier Transform Infrared Spectroscopy 

The Fourier transform infrared (FTIR) spectra of SFBP, HFBP, SFBP-H and HFBP-H were analyzed using FTIR spectroscopy. The sample was mixed with potassium bromide and analyzed according to Elavarasan et al. [28]. Spectra were obtained from 4000 to 400 cm^−1^ with an average of 32 automated scans observed at a resolution of 4 cm^−1^.

### 2.8. Determination of Amino Acid Composition

The total amino acid (TAA) composition was measured by an Agilent 1100 series HPLC system (Agilent Technologies, Inc., Santa Clara, CA, USA) equipped with a C18 ODS Hypersil column (4.6 mm × 250 mm i.d., 5 μm particle size; Agilent Technologies, Inc.). According to Liu et al. [9], the samples were hydrolyzed in 6 M HCl under vacuum at 110 °C for 22 h in sealed tubes. Amino acid analysis was conducted using pre-column derivatization with o-phthalaldehyde and fluorenylmethyl chloroformate. The detection wavelength was 338 nm, except for hydroxyproline, which was detected at 262 nm. For the determination of free amino acid (FAA) composition, the hydrolysates at different time intervals of hydrolysis were mixed with the same volume of 15% trichloroacetic acid (TCA) to collect the supernatant. The FAA profile was then analyzed with an automatic amino acid analyzer (Sykam S-433D, Berchtesgaden, Germany).

### 2.9. Determination of Molecular Weight Distribution

The molecular weight (MW) distribution of peptides was examined on a Waters e2695 HPLC system equipped with a TSKgel G2000 SW_XL_ column (7.8 mm × 300 mm, Tosoh, Tokyo, Japan) and a TSKgel SW_XL_ guard column (6.0 mm × 40 mm, Tosoh, Tokyo, Japan). The chromatographic conditions were as follows, according to Zhang et al. [17]: the eluent was 45% acetonitrile containing 0.1% (*v*/*v*) trifluoroacetic acid (TFA), the flow rate was 0.5 mL/min, the injection volume was 10 μL and the absorbance was monitored at 220 nm. The standards used for the molecular weight calibration were as follows: HHL (429 Da), bacitracin (1422 Da), aprotinin (6.5 kDa) and cytochrome C (12.4 kDa). 

### 2.10. Determination of Antioxidant Activity of Hydrolysates

#### 2.10.1. DPPH Radical Scavenging Activity

The DPPH radical scavenging activity of the hydrolysates was determined according to Saisavoey et al. [29], with some modifications. Briefly, 1.0 mL protein hydrolysate at different hydrolysis times was added to 1.0 mL of 0.15 mmol/L DPPH solution in 95% ethanol. The mixture was left in the dark for 30 min at room temperature and then its absorbance was measured at 517 nm. The scavenging effect for DPPH radical was expressed as follows:

DPPH radical scavenging activity (%) = [1 − (As − Ab)/Ac] × 100(1)

where As represents the absorbance of a sample at 517 nm, Ab represents the absorbance of a sample blank without DPPH, and Ac represents the absorbance of the control without a sample.

#### 2.10.2. ABTS Radical Scavenging Activity

The ABTS radical scavenging activity of the hydrolysates was determined according to Zheng et al. [30], with some modifications. ABTS radical stock solution was prepared by mixing 7 mmol/L ABTS with 140 mmol/L potassium persulfate, kept in the dark at room temperature for 12–16 h. The ABTS solution was diluted with phosphate buffer (0.2 M, pH 7.4) to an absorbance of 0.70 ± 0.02 at 734 nm. A total of 50 μL of sample was added to 150 μL of diluted ABTS solution, followed by incubation at room temperature for 6 min. The absorption was monitored at 734 nm. The scavenging effect for ABTS radical was expressed as follows:

ABTS radical scavenging activity (%) = [1 − (As − Ab)/Ac] × 100(2)

where As represents the absorbance of the sample at 734 nm, Ab represents the absorbance of the sample blank without ABTS, and Ac represents the absorbance of the control without sample.

### 2.11. Statistical Analysis

The results were expressed as mean ± standard deviation (SD) of triplicates. Analysis of variance (ANOVA) was conducted using SPSS 21.0 software (SPSS Inc., Chicago, IL, USA). Significant differences between means were identified using Duncan’s multiple range tests (*p* < 0.05).

## 3. Results and Discussion

### 3.1. Proximate and Amino Acid Compositions of FBP

The crude protein contents were 81.09 and 84.88 g/100 g on a dry basis, for SFBP and HFBP, respectively. Such levels of protein were also reported by Jeya Shakila et al. [31]. Compared to fish skin, fish bone usually contains lower protein, due to the presence of higher ash and fat contents [32]. The extraction rate of protein recovery from fish backbone was about 54% for both the samples, which was similar to that of cod bone gelatin (60%) [20], but higher than that of Nile perch (*Lates niloticus*) bone gelatin (<40%) [32]. The recovered protein had ash contents with values of 15.15 and 13.51 g/100 g for SFBP and HFBP, respectively. The results were comparable to the hydrolysates from Atlantic codfish (*Gadus morhua*) frames (15 g/100g) [12], but were slightly higher than the gelatin prepared from red snapper (*Lutjanus campechanus*) bone (10.32 g/100 g) [31]. The high ash contents in the recovered bone protein were most likely from an inadequate decalcification process and high mineral contents in the bones [31]. Actually, proteins with high mineral contents might also show special functional properties. A recent study found that high-mineral-content (>20%) gelatin, prepared from saithe (*Pollachius virens*) skin, exhibited higher viscoelastic behavior and foaming properties compared with commercial gelatin, which might lead it to find applications in cosmetics and foods sectors, as a potential new microencapsulating agent [33]. 

The fish backbones that were used in the present study did not undergo decalcification steps before the hydrothermal pretreatment. Compared with traditional alkali/acid extraction methods, hydrothermal pretreatment, by steam explosion-assisted extraction and hot-pressure extraction, was a promising process to recover protein from fish backbones, and could avoid the disadvantages of the decalcification procedure. In our previous study, no significant difference in protein recovery from fish scales was found between the hydrothermal pretreatment with or without decalcification [17]. Further studies should examine whether decalcification is actually necessary for achieving high protein recovery and good functional properties. Except for the thermal effect, as in hot-pressure extraction, there is a physical tearing effect during the steam explosion process, to overcome the key barriers limiting the degradation and extraction of the biomass [23]. As a result, similar amounts of protein could be extracted from the exploded fish bone, at a relatively low temperature. 

The TAA contents of FBP treated by SE and HPE pretreatment, were 749.7 and 833.7 mg/g FBP, respectively. The amino acid composition of FBP samples per 1000 total residues is shown in Table 1. Glycine was the most abundant amino acid in both the samples, making up around a third of the total residues. They were also rich in alanine (about 123 residues), proline (90.9–101.1 residues), glutamic acid (85.1–90.2 residues), hydroxyproline (69.7–82.2 residues), aspartic acid (54.2–57.3 residues), and arginine (52.5–53.3 residues), but poor in histidine and tyrosine residues. Similar results were also found in gelatin hydrolysates from salmon bone [29], *Thunnus orientalis* bone [34], and barred mackerel byproducts [35]. The TAA profiles of the recovered proteins were very similar to those of type-I collagen isolated from bighead carp bones [9]. However, slight differences in TAA compositions were also found between SFBP and HFBP. Proline and hydroxyproline (imino acid) accounted for ~23% of the amino acid content of the collagen molecule [36]. In the present study, imino acid residue totaled 183.3 per 1000 total residue in SFBP, which was approximately 1.14 times higher than that of the HFBP (160.6). Accordingly, the content of imino acids contributes to the thermal stability of collagens [9]. The content of imino acid in SFBP was similar to that of collagen extracted from tilapia scales, through extrusion–hydro-extraction (187.7) [37], but higher than that of pepsin-solubilized collagen from bighead carp bones (174) and swim bladders (175), through traditional acid/alkali extraction [9]. These results reflect the high effectiveness of steam explosion-assisted extraction, for the release of proline and hydroxyproline from the native fish backbone materials. 

In the FBP, the contents of histidine, threonine, cysteine + methionine, valine, phenylalanine + tyrosine, isoleucine, leucine, and lysine were 4.2 and 6.5 mg/g, 10.7 and 14.4 mg/g, 57.7 and 64.9 mg/g, 17.5 and 21.1 mg/g, 18.5 and 24.2 mg/g, 10.5 and 13.7 mg/g, 20.3 and 25.5 mg/g, 25.4 and 29.5 mg/g, respectively. All the essential amino acids (EAAs) totaled 17.50% and 19.57% in the total amino acids for SFBP and HFBP, respectively. In spite of the low levels of some EAAs, the FBP may be used as a food additive, to improve the functional properties of protein gel [4,38]. Moreover, many studies have found a good correlation between certain amino acid residues and the bioactivity of food-borne hydrolysates. The antioxidant activity of peptides is mostly dependent on the composition and sequence of amino acids [29]. Accordingly, glycine and hydrophobic amino acids (alanine, proline, hydroxyproline, valine, and leucine) are key factors for potential antioxidant activity, by improving the scavenging effect of hydrolysates on free radicals [35,39]. A recent study showed that several antioxidant small peptides, such as glycine-proline-proline (GPP) and glycine-alanine-alanine (GAA), are encrypted in the amino acid sequences of salmon viscera extracts [40]. Furthermore, acidic amino acids, such as glutamic acid and aspartic acid, can quench unpaired electrons and free radicals, by providing protons [41]. In the present study, the recovered proteins, by hydrothermal pretreatment, might be a good source when seeking to extract bioactive ingredients, due to the high levels of certain amino acid residues, such as glycine, alanine, and proline.

### 3.2. DH and NR

**Figure 1:**
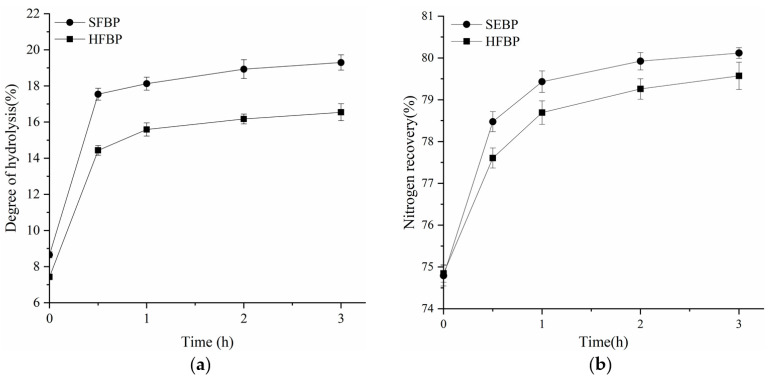
Degree of hydrolysis (**a**) and nitrogen recovery (**b**) of SFBP and HFBP by Flavourzyme. SFBP: fish bone protein prepared through steam explosion-assisted extraction; HFBP: fish bone protein prepared through hot-pressure extraction.

DH can be used as an indicator of the break of peptide bonds, whereas NR reflects the ability to recover peptides from the hydrolysis process [20]. The DH and NR of fish bone protein, treated with Flavourzyme, increased with the prolonged hydrolysis time (Figure 1). The initial DH values of 8.65% and 7.43% at 0 h indicated that some protein molecules had been partially hydrolyzed to peptides or free amino acids, during the SE and HPE pretreatment. The DH and NR of SFBP and HFBP both increased sharply (*p* < 0.05) during the first 0.5 h, and then showed a slow increase. With the increase in hydrolysis time, a large amount of soluble peptide was generated, resulting in higher NR values. After 3 h of hydrolysis, the NR values of SFBP and HFBP samples finally reached 80.12% and 79.57%, respectively. The hydrolysis curves were similar to those of protein hydrolysates from cod bone [20] and thornback ray [42]. Nevertheless, the values of DH and NR in this study were higher than that of the protein recovered from cod bone, which was treated at 121 °C for 90 min, followed by Flavourzyme and Trypsin hydrolysis [20]. The differences in enzymatic extraction between SFBP and HFBP, were found to be mainly due to the differences in the protein structure of the substrate. More significantly, how the pretreatment method affects the protein structure and enzymatic hydrolysis needs to be studied further.

### 3.3. FTIR 

**Figure 2:**
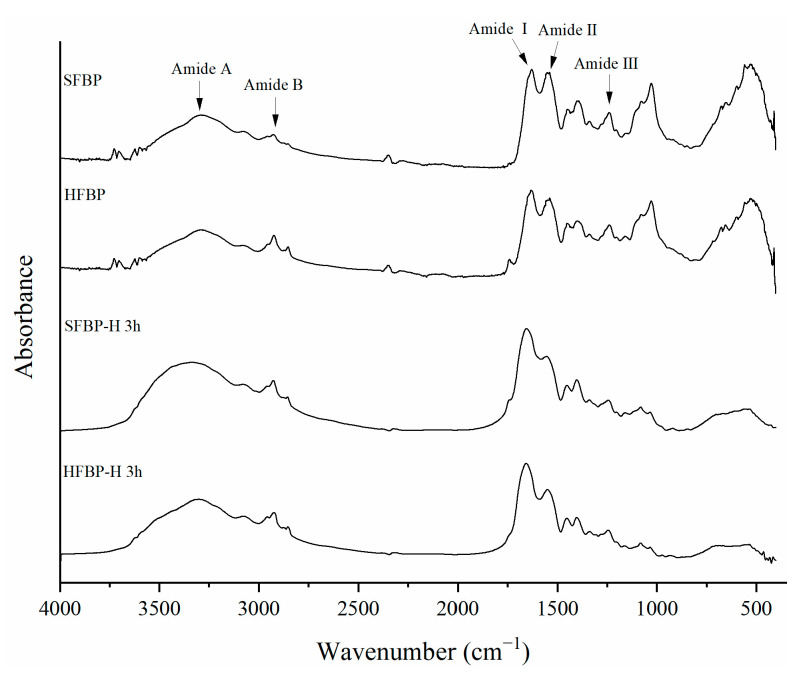
FTIR spectra of SFBP, HFBP, SFBP-H and HFBP-H. SFBP: fish bone protein prepared through steam explosion-assisted extraction; HFBP: fish bone protein prepared through hot-pressure extraction; SFBP-H: SFBP hydrolysates; HFBP-H: HFBP hydrolysates.

FTIR spectroscopy can be used to characterize the changes of functional groups and secondary structures of protein. As shown in Figure 2, all the samples exhibited typical amide vibrations at amide A, amide B, amide I, amide II, and amide III, with similar spectral patterns, indicating their similar chemical compositions. The amide A region, at 3292−3340 cm^−1^, was found in all the samples, representing the N–H stretching vibration coupled with hydrogen bonding [28]. SFBP showed a higher amplitude than HFBP, suggesting greater free amino groups were caused by the SE pretreatment. The amide I region (1600–1700 cm^−1^), involving mainly the C=O stretching vibration, was found at 1628 cm^−1^ and 1633 cm^−1^ for the SFBP and HFBP samples, respectively, which was indicative of the presence of a *β*-sheet secondary structure in the recovered bone proteins. This phenomenon might be attributed to the disruption of the native structure in bone materials during the SE and HPE pretreatment process. After enzymatic hydrolysis, a shift towards higher wavenumbers was found in the peak at 1654 cm^−1^ and 1657 cm^−1^, for the SFBP-H and HFBP-H samples, respectively, indicating the disruption of some ordered *β*-sheet structures. 

The amide III region (1220–1340 cm^−1^) is associated with C–N stretching vibrations, and O=C–N and N-H bending. Due to atmospheric water vapor interference in the amide I region of FTIR, the deconvolution of the amide III region is recommended for more accurate analysis of the protein secondary structure [26]. The relative contents of the microstructural components could be obtained based on the *β*-sheet (1220–1250 cm^−1^), random coil (1250–1270 cm^−1^), *β*-turn (1270–1295 cm^−1^), and *α*-helix (1295–1330 cm^−1^) [26]. As shown in Table 2, the secondary structures of protein in the SFBP and HFBP samples were mainly *β*-sheet (about 52%) and random coil (about 33%). It is widely known that the main structure of the collagen molecule in native collagen materials is a triple superhelix [43]. Thus, the transformation of an *α*-helix to a random coil structure is related to an employed pretreatment that causes a higher disorder of the molecular structure [33]. Interestingly, the contents of the *β*-sheet reduced to 41.72% and 35.62%, respectively, and the contents of the *β*-turn increased to about 20% in both the SFBP-H and HFBP-H samples, after 3 h of hydrolysis. This was consistent with the results obtained from amide I. It is worth noting that the percent of *β*-sheets, before enzymatic hydrolysis, seemed similar for both the samples that underwent the different pretreatments, while after enzymatic hydrolysis, these values showed an obvious difference. The difference may result from the variance of the cleavage sites exposed during the SE and HPE processes, which requires more study.

### 3.4. Molecular Weight Distribution of FBP Hydrolysates

**Figure 3:**
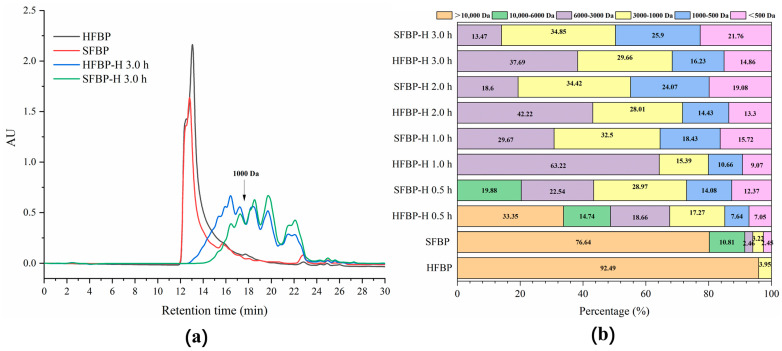
Distribution of molecular weights of SFBP-H and HFBP-H. (**a**) The molecular weight distribution of bone protein and bone hydrolysates. (**b**) Fractions of the chromatogram based on molecular weights of SFBP-H and HFBP-H. SFBP: fish bone protein prepared through steam explosion-assisted extraction; HFBP: fish bone protein prepared through hot-pressure extraction; SFBP-H: SFBP hydrolysates; HFBP-H: HFBP hydrolysates.

The molecular weight distribution of FBP, hydrolyzed by Flavourzyme at different times, is shown in Figure 3. The samples were divided into six fractions (>10,000 Da, 10,000–6000 Da, 6000–3000 Da, 3000–1000 Da, 1000–500 Da, and <500 Da), by gel filtration chromatography. Interestingly, there was a difference in the molecular weight distribution, even before enzymatic hydrolysis. Compared to the hydrolysates, the majority of the protein recovered from fish bone ranged above 10,000 Da, with values of 76.44% and 92.49% for the SFBP and HFBP samples, respectively. When Flavourzyme was added to the samples, FBP was constantly decomposed into low-molecular-weight peptides. After 0.5 h of hydrolysis, the proportions of SFBP and HFBP hydrolysates with a molecular weight below 6000 Da, were 80.12% and 51.91%, respectively. The hydrolysates with higher DH showed a higher proportion of low-molecular-weight peptides, as demonstrated by Chiang et al. [44]. The content of the components that were below 3000 Da in 3-h hydrolysate from the SFBP sample was 86.53%, which was about 1.38 times higher than that of the HFBP hydrolysates. Under the same hydrolysis conditions, the fractions of small peptides, below 1000 Da, in the SFBP and HFBP hydrolysates were 47.66% and 31.07%, respectively. These differences might be largely due to the structural differences of the substrates for enzymatic hydrolysis, which were mainly caused by the pretreatment methods. The experiments indicated that SE pretreatment might expose more enzyme sites to accelerated peptide release during the hydrolysis reaction. 

### 3.5. Free Amino Acid Composition of FBP Hydrolysates 

**Figure 4:**
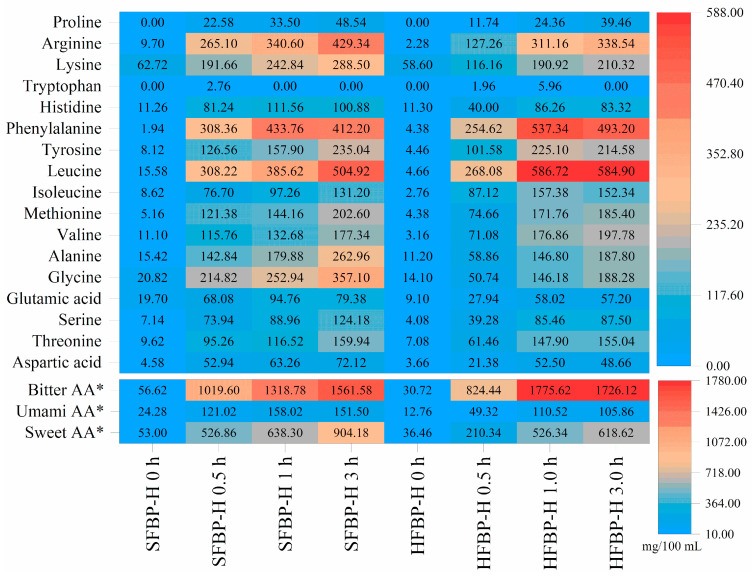
Heatmap of FAAs’ composition (mg/100 mL) and major taste components of FBP hydrolysates. SFBP: fish bone protein prepared through steam explosion-assisted extraction; HFBP: fish bone protein prepared through hot-pressure extraction; SFBP-H: SFBP hydrolysates; HFBP-H: HFBP hydrolysates. * Bitter AA: calculated from the sum of valine, isoleucine, leucine, phenylalanine, histidine, tryptophan and tyrosine; Sweet AA: calculated from the sum of threonine, serine, glycine and alanine; Umami AA: calculated from the sum of aspartic acid and glutamic acid.

A heatmap of the FAAs’ composition in FBP hydrolysates at different hydrolysis times is shown in Figure 4. Most of the FAA contents increased with the increase in hydrolysis time. When hydrolyzed for 3 h, the amounts of total FAAs increased dramatically, from 211.48 and 145.20 mg/100 mL to 3586.24 and 3224.32 mg/100 mL, for the SFBP and HFBP hydrolysates, respectively. Before the enzymatic hydrolysis, the contents of most FAAs of SFBP were higher than those of HFBP, except for phenylalanine, tryptophan, and proline. The dominant FAAs of all the hydrolysates were leucine, phenylalanine, and arginine, followed by glycine and lysine for the SFBP hydrolysates, while the top five FAAs in the HFBP hydrolysates were leucine, phenylalanine, arginine, tyrosine, and lysine. Compared with HFBP, more glycine and alanine residues were released from SFBP after the hydrolysis by Flavourzyme. This indicates that more cleavage sites were generated after SE pretreatment compared with the HPE process.

The composition and content of FAAs have a major impact on the flavor properties, directly or indirectly [45]. Flavourzyme displays a great advantage in improving the flavor of protein hydrolysate, by releasing monosodium glutamate-like amino acids [46]. The contents of umami amino acids (glutamic acid and aspartic acid) in the SFBP and HFBP hydrolysates increased by 6.24 and 8.30 times, respectively, after Flavourzyme hydrolysis for 3 h. Remarkably, the SFBP hydrolysates had a significantly (*p* < 0.05) higher level of umami and sweet amino acids than the HFBP hydrolysates did, during the whole hydrolysis.

For both the FBP hydrolysates, bitter amino acids were the most abundant, followed by sweet and umami amino acids. Bitter amino acids occupied 43.54% of the total FAAs in the SFBP hydrolysates, while the total amount of umami and sweet amino acids accounted for 29.44%. Similarly, for the HFBP hydrolysates, bitter amino acids account for 53.53%, while the total of umami and sweet amino acids account for 22.47%. Furthermore, the ratio of bitter/umami components in the SFBP hydrolysates at 3 h was 10.31, whereas that in the HFBP hydrolysates was 16.31. These results indicate that SE pretreatment and Flavourzyme hydrolysis can produce a collagen hydrolysate with less bitterness and umami taste. In a previous study, protein hydrolysates with low bitterness could be obtained from Alaska pollock frame-treated by a hot-pressure process, followed by mixed enzymes for animal proteolysis, Protamex or Flavourzyme [47]. In the present study, further sensory evaluation was needed to confirm the phenotype flavor of the bone hydrolysates.

### 3.6. Antioxidant Activities of FBP Hydrolysates

Antioxidant compounds play an important role in the prevention of free radical-induced tissue damage [48]. Compared with some synthetic compounds, such as butylated hydroxyanisole (BHA) and butylated hydroxytoluene (BHT), food-derived antioxidant peptides are potential antioxidants with fewer negative side effects [49]. Recently, a series of protein hydrolysates, with in vitro antioxidant activity, has been discovered from fish byproducts, such as yak (*Bos grunniens*) bone [41], *Cyprinus carpio* skin [50], tilapia (*Oreochromis nilotica* L.) skin [51], salmon (*Salmo salar*) trimmings [52], and rainbow trout (*Oncorhynchus mykiss*) byproducts [53].

**Figure 5:**
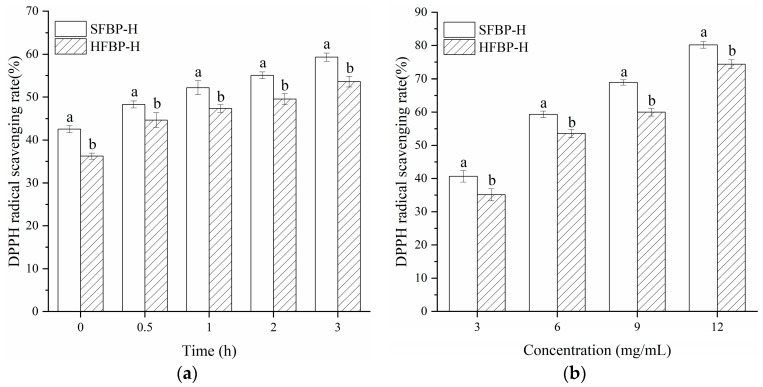

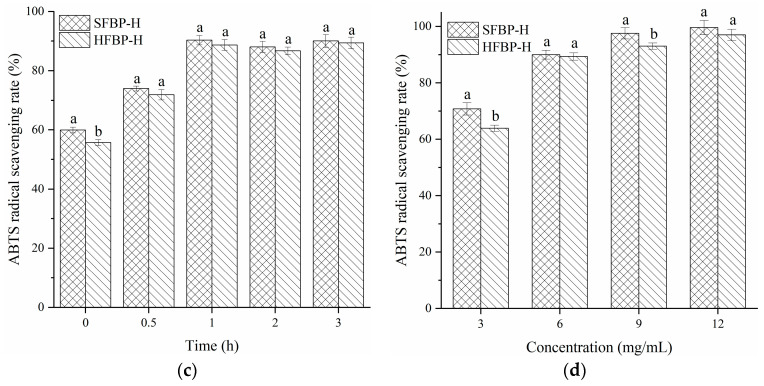
Antioxidant activities of SFBP-H and HFBP-H. DPPH radicals scavenging activity (**a**) and ABTS radicals scavenging activity (**c**) of SFBP-H and HFBP-H treated with Flavourzyme for various hydrolysis times (0–3 h); the concentration of all hydrolysates used in the experiments was 6 mg/mL. DPPH radicals scavenging activity (**b**) and ABTS radicals scavenging activity (**d**) of SFBP-H and HFBP-H at various concentrations after hydrolysis for 3 h. SFBP: fish bone protein prepared through steam explosion-assisted extraction; HFBP: fish bone protein prepared through hot-pressure extraction; SFBP-H: SFBP hydrolysates; HFBP-H: HFBP hydrolysates. Different letters indicate that the results of SFBP-H and HFBP-H differ significantly (*p* < 0.05).

DPPH radical scavenging activities are one of the most used methods to evaluate the ability of antioxidants to scavenge free radicals, while an ABTS radical elimination assay measures the antioxidant activity of both hydrophilic and lipophilic compounds [13]. The scavenging effect of bone hydrolysates on DPPH and ABTS radicals is shown in Figure 5. The SFBP samples showed higher scavenging activities for the DPPH and ABTS radicals (42.56% and 59.94%) before enzymatic hydrolysis than that of the HFBP samples (36.24% and 55.71%). This indicated that some peptides with high antioxidant activity were released under hydrothermal conditions, even without enzymatic hydrolysis. Similar phenomena were also found in our previous study about gelatin hydrolysates that were prepared from tilapia (*Oreochromis niloticus*) scale, through hydrothermal pretreatment [17]. 

As shown in Figure 5a, the DPPH radical scavenging activity of SFBP hydrolysates increased with hydrolysis time, and the same trend was also found in HFBP hydrolysates. The scavenging activities of both the bone hydrolysates reached maximum values of 59.34% and 53.58% at 3 h, respectively. The HFBP hydrolysates presented a lower DPPH value than that of the SFBP hydrolysates. All the samples exhibited DPPH radical scavenging activity in a dose-dependent manner (Figure 5b). The IC_50_ values of SFBP-H and HFBP-H were 4.24 and 5.33 mg/mL, respectively. The IC_50_ values of the protein hydrolysates were comparable to those of the hydrolysates obtained from *Cyprinus carpio haematopterus* scale gelatin (IC_50_ 4.46 and 6.97 mg/mL) [54], but lower than that of the hydrolysates from shark skin gelatin (IC_50_ 27.39 mg/mL) [55]. As shown in Figure 5c, the ABTS radical scavenging activity of SFBP-H and HFBP-H also increased with hydrolysis time, during the first 1 h, which finally reached about 90.02%, while it changed insignificantly (*p* > 0.05) at a longer hydrolysis time. The IC_50_ values of SFBP-H and HFBP-H were 1.93 and 2.28 mg/mL, respectively (Figure 5d). The results were lower than those of pepsin-solubilized collagen hydrolysate from mackerel (*Scomber japonicus*) bone and skin, at 250 °C and 70 bar (2.61 and 2.50 mg/mL, respectively) [19]. 

In the present study, all the bone hydrolysates showed a better ability to scavenge ABTS than DPPH radicals, which was consistent with the results from Lima et al. [13]. The antioxidant activity of protein hydrolysates could be influenced by the size, quantity, composition, and sequence of low-molecular-weight peptides [56]. Comparatively speaking, SFBP and its hydrolysates might have more power, acting as hydrogen donors, to terminate the radical chain reaction, thus scavenging free radicals. However, these are in-vitro methods, and subsequent analyses are necessary.

## 4. Conclusions

Steam explosion-assisted extraction was an effective method for extracting fish bone protein. The FBP contains a high content of protein, and is rich in glycine, alanine, proline, and hydroxyproline. Both nitrogen recovery and free amino acids of the hydrolysates increased with the degree of hydrolysis of FBP, which was hydrolyzed by Flavourzyme. Compared with HFBP hydrolysates, the SFBP hydrolysates mainly distributed below 3000 Da, with stronger free radical scavenging activities, and had higher levels of umami and sweet free amino acids. Steam explosion-assisted extraction is a promising route to recover proteins from fish backbone and facilitate the enzymatic release of antioxidant peptides. The functionalities and flavor characteristics of protein hydrolysates will be further studied. The recovered proteins, especially bioactive peptides, can be used as supplements in food, cosmetic and pharmaceutical industries. Moreover, steam explosion equipment with a 10 m^3^ biomass chamber has been developed, increasing the potential of SE for industrial applications.

## Figures and Tables

**Table 1:** Amino acid composition of fish bone protein treated by steam explosion-assisted extraction and hot-pressure extraction (results are expressed as residues/1000 total residues).

Amino Acids	SFBP	HFBP
Aspartic acid/asparagine	54.2	57.3
Glutamic acid/glutamine	85.1	90.2
Serine	27.1	30.9
Histidine	4.2	5.9
Glycine	333.5	328.5
Threonine	20.8	21.7
Arginine	53.3	52.5
Alanine	123.4	123.1
Tyrosine	2.7	3.9
Valine	23.3	25.3
Methionine	11.2	13.6
Phenylalanine	14.4	16.3
Isoleucine	12.5	14.7
Leucine	24.1	27.3
Lysine	27.0	28.3
Proline	101.1	90.9
Hydroxyproline	82.2	69.7
Total	1000	1000
Imino acids	183.3	160.6

SFBP: fish bone protein prepared through steam explosion pretreatment; HFBP: fish bone protein prepared through hot-pressure extraction pretreatment.

**Table 2:** The relative contents of the secondary structure (%) of fish bone protein before and after enzymatic hydrolysis.

Samples	*β*-Sheet/%	Random Coil/%	*β*-Turn/%	*α*-Helix/%
SFBP	52.14	33.32	14.54	-
HFBP	51.40	33.36	11.88	3.35
SFBP-H	41.72	34.13	19.99	4.17
HFBP-H	35.62	36.46	20.85	7.07

SFBP: fish bone protein prepared through steam explosion-assisted extraction; HFBP: fish bone protein prepared through hot-pressure extraction; SFBP-H: SFBP hydrolysates; HFBP-H: HFBP hydrolysates.

## Data Availability

Data are available upon request.
